# Facile large-area uniform photolithography of membrane diffractive lens based on vacuum assisted self contact method

**DOI:** 10.1038/s41598-020-65990-2

**Published:** 2020-06-02

**Authors:** Guohan Gao, Lihua Wang, Heng Shi, Dun Liu, Bin Fan, Chunlin Guan

**Affiliations:** 1 Institute of Optics and Electronics, Chinese Academy of Sciences, Chengdu, 610209 China; 2 University of Chinese Academy of Sciences, Beijing, 100049 China

**Keywords:** Applied optics, Materials for optics, Nanoscience and technology

## Abstract

Optical polyimide (PI) membrane is a promising substrate material for diffractive lens applied in future large-aperture space based imaging system because of its light weight, environmental adaptability and deployable feature. In this letter, we put forward a facile large-area uniform photolithography technique using vacuum assisted self contact method to fabricate large-aperture membrane diffractive lens. We fabricated a φ 400 mm aperture membrane off-axis 2-levels Fresnel Zone Lens (FZL) based on the method and achieved uniformly distributed photoresist morphology as well as over 36.6% average diffraction efficiency in full aperture. The results demonstrated that vacuum assisted self contact method effectively eliminates considerable air gaps caused by unevenness of large area photomask and substrate, thus facilitates uniform light field distribution in photoresist. This work provides reference to fabrication techniques of large aperture membrane diffractive lens, and offers feasible methods for future large area flexible electronics manufacturing.

## Introduction

Very large aperture diffractive lens, a critical optical element applied in next generation space based imaging system, becomes increasingly attractive because of the relaxed surface figure tolerance, higher foldable ratio and light weight^1^. Owing to advancement of polyimide (PI) material research, PI membrane with good space environmental adaptability, micro-fabrication compatibility and good optical property became a potential choice for membrane diffractive lens substrate on which diffractive patterns are fabricated so that a membrane diffractive lens is formed and acts as the primary lens in the optical imaging system. The adoption of optical PI membrane material instead of thin glass material can further decrease weight and increase vibration enduring capability^2,3^. Sub-meter scale Fresnel Zone Lens (FZL) is a widely adopted diffractive pattern in stitched PI membrane primary lens design due to its mature achromatic model and relatively lower fabrication precision requirement^4^. However, fabricating micron scale patterns on such large area flexible substrate is challenging for existing fabrication capability. Lawrence Livermore National Laboratory (LLNL) adopted two-step method that firstly a master silica template is made by direct laser writing (DLW) and ion beam etching (IBE) and secondly a PI membrane with identical structure is replicated through casting and curing^5^. Institute of Optics and Electronics (IOE) and Changchun Institute of Optics, Fine Mechanics and Physics (CIOMP) adopted similar fabrication method and accumulated useful experiences^6–8^. The biggest challenge for the two-step method is that separating PI membrane from silica master template causes dimensional change and that mounting PI membrane by stretching and fixing causes additional dimensional change, which finally lead to distortion of diffractive pattern and failure of imaging^9^. To avoid the pattern distortion problem, we can also start fabrication of PI membrane FZL with membrane stretching and fixing then sculpture diffractive patterns on the already mounted PI membrane^10^. Photolithography and Nano-imprinting are capable of wafer-scale high resolution pattern transfer process and are extensively applied in semiconductor field and flat display industry^11–15^. However, large aperture membrane FZL is much larger in substrate size and features flexible rather than rigid in substrate material. Simply applying existing photolithography and nano-imprinting techniques hardly meet fabrication requirement mainly because commercial lithographic machines are not designed for such application. A common challenge for large area photolithography and nano-imprinting is poor uniformity caused by non-uniform air gap distance between photomask and substrate or between template and substrate respectively. The underlying reason for non-uniform large area photolithography is the unevenness of photomask and substrate, and factors influencing unevenness of photomask were systematically studied^16,17^.

In this letter, we put forward a vacuum assisted self contact photolithography method and fabricated a φ 400 mm aperture PI membrane off-axis FZL as a segmented primary lens based on this method and achieved good uniformity over full aperture. We discussed underlying mechanism through theoretical analysis and computer simulation, and demonstrated feasibility of this method in fabricating large area uniform membrane diffractive lens.

## Materials and methods

**Figure 1:**
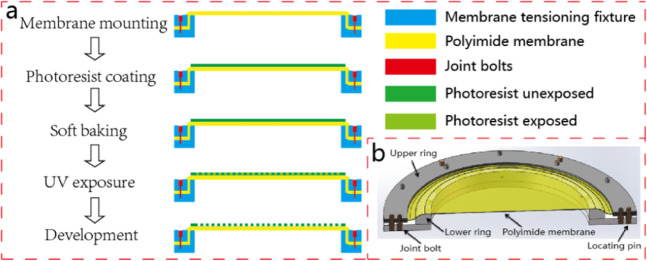
Process flow of PI membrane diffractive lens fabrication, in which conventional contact and vacuum assisted self contact photolithography share the same membrane mounting, photoresist coating, soft baking and development process while ultra-violet (UV) exposure process is different (**a**). Membrane tensioning fixture, which consists of upper ring, lower ring, joint bolt and locating pin (**b**).

Optical PI membranes were synthesized by 1,2,4,5-Benzenetetracarboxylic anhydride (PMDA) and 2,2’-Bistrifluoromethylbenzidine, and formed uniform membranes with 25 μm thickness and Φ 400 mm aperture by multiple spin cast and imidization. Then off-axis FZL pattern was fabricated on PI membrane surface through photolithography process shown in Fig. 1(a). PI membrane was stretched and mounted on membrane tensioning fixture shown in Fig. 1(b).

**Figure 2:**
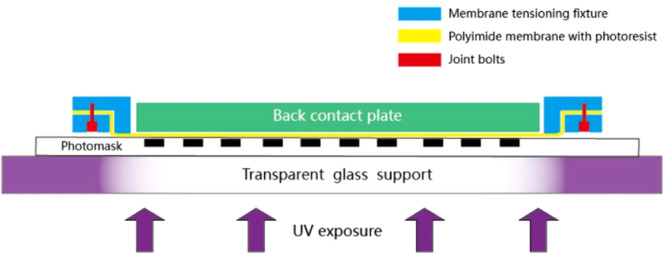
Principle of conventional contact photolithography without vacuum. Photomask is supported by a transparent glass made of high quality fused silica. Ultraviolet light source is underneath. Back contact plate should have good flatness which is normally smaller than 2 μm in peak to valley.

During photolithography PI membrane experienced surface cleaning, photoresist coating and prebaking procedures before it comes to UV exposure. We compared conventional contact photolithography and vacuum assisted self contact photolithography, the schematic of conventional one is shown in Fig. 2 below.

Before UV exposure, PI membrane coated with photoresist is placed face down and sandwiched between large area photomask and back contact plate. During UV exposure, make sure that there is no movement among photomask, membrane and back contact plate.

**Figure 3:**
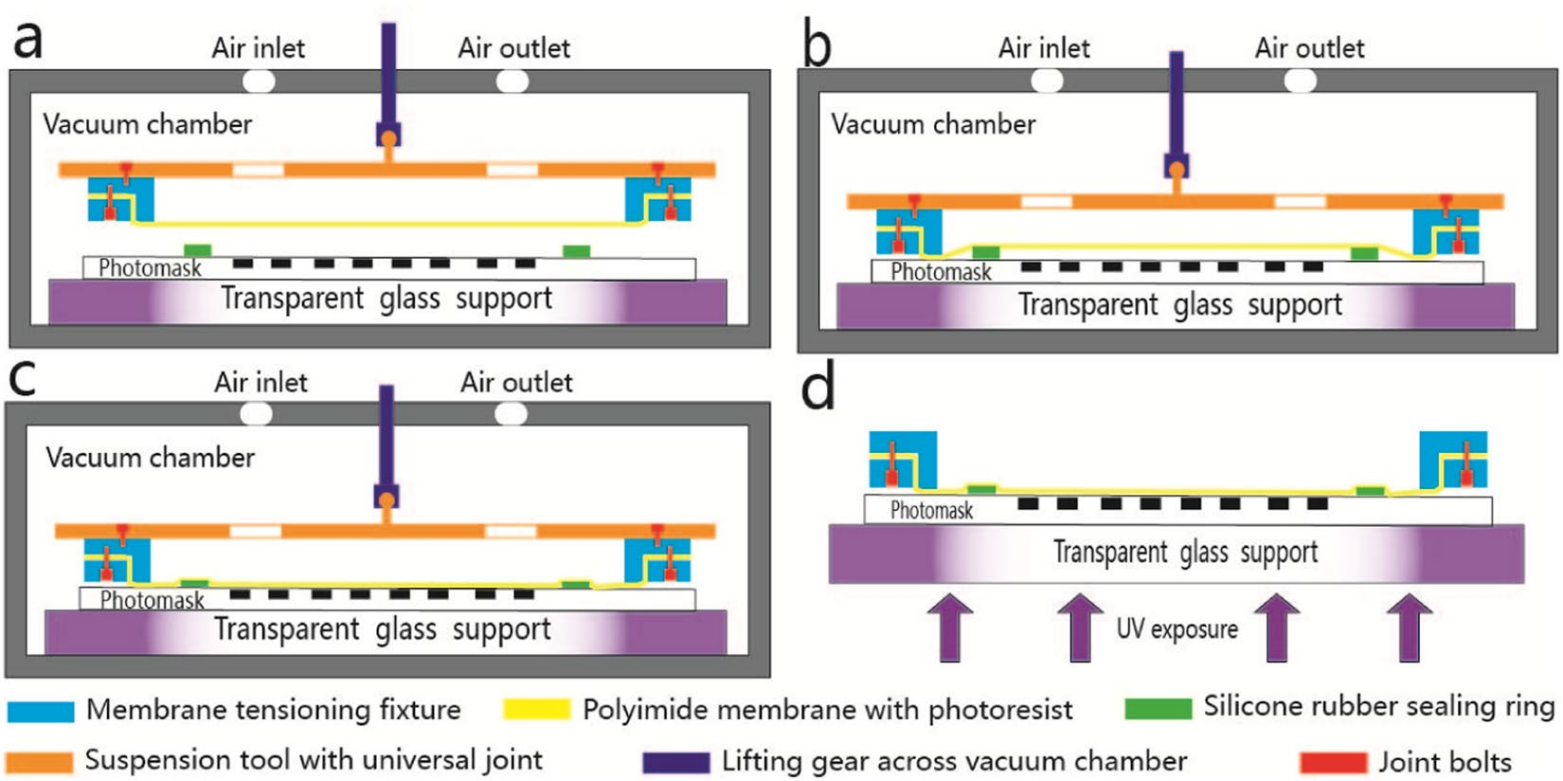
Vacuum assisted self contact photolithography 1st step (**a**), 2nd step (**b**), 3rd step (**c**) and 4th step (**d**). The first three steps are conducted inside vacuum chamber, the final step is conducted outside.

There are four steps in vacuum assisted self contact photolithography method shown in Fig. 3. For the first step shown in Fig. 3(a), mounted PI membrane coated with photoresist is connected to suspension tool with universal joint hanging to lifting gear across vacuum chamber and silicone rubber sealing ring is placed on photomask surface outside of pattern area with sufficient distance (normally larger than 5 mm) kept between silicone rubber sealing ring and membrane surface. Then close chamber door and turn on vacuum pump. For the second step shown in Fig. 3(b), mounted PI membrane is lowered down and pressed to contact silicone rubber sealing ring with controlled force by lifting gear across vacuum chamber when vacuum degree reached set point. Key point in this step is to make sure all parts of silicone rubber sealing ring are in good contact with photomask and PI membrane thus an enclosed space is formed within membrane, photomask and silicone rubber sealing ring. For the third step illustrated in Fig. 3(c), turn off vacuum pump and revert to atmospheric pressure. The enclosed space is compressed by pressure difference facilitating full contact between membrane and photomask. For the final step shown in Fig. 3(d), membrane tensioning fixture is detached from suspension tool and taken out carried by transparent glass support then experienced uniform UV exposure. Detailed process parameters for both conventional contact and vacuum assisted self contact photolithography are shown in Table 1 below.

After UV exposure, it is necessary to break the vacuum between membrane and photomask in order to peel off membrane before development process. We managed to break the vacuum by inserting some thin sheet such as A4 paper between silicone rubber sealing ring and photomask to let air in.

**Table 1:** Detailed process parameters.

Process Steps	Parameters
Photoresist^a^	AZ3100, 1000 rpm, 90 s
Soft bake	100 °С, 10 min
Exposure	4.5 mW/cm^2^, 25 s
Development	5%TMAH, 45 s

^a^AZ3100 is a commercial positive photoresist.

## Results and discussion

### Conventional contact photolithography

**Figure 4:**
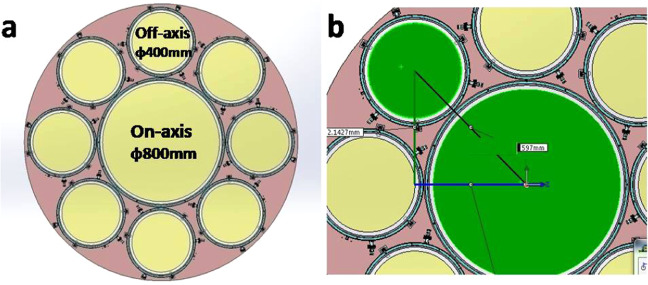
Schematic of primary lens mentioned in Table 2 consists of 8 identical off-axis FZLs with 400 mm aperture and 1 on-axis FZL with 800 mm aperture (**a**), distance from primary lens center to off-axis FZL center is 597 mm (**b**).

The optical parameters for the membrane off-axis FZL are shown in Table 2 below. It is important to notice that the primary lens of 1500 mm diameter consists of 8 identical off-axis FZLs and 1 on-axis FZL shown in Fig. 4 below. The central on-axis FZL is around 800 mm in diameter and edge off-axis FZL is round 400 mm in diameter. In this work, our fabrication and characterization focus on one of 400 mm aperture off-axis FZLs. Because any line and space in off-axis FZL is an arc with radius of curvature ranging from 350 mm to 750 mm, it looks like binary grating in microscopic view.

**Table 2:** Optical parameters for the primary lens and membrane off-axis FZL.

Items	Values
Aperture of primary lens	Φ 1500 mm
Aperture of each off-axis FZL	Φ 400 mm
Central wavelength (λ)	600 nm
F number	7
Focal length	10.5 m
Smallest pitch	8.4 μm
Number of levels	2
Refractive index (n)	1.66
Structure depth^a^	(2k + 1)(n − 1)^−1^λ/2
Theoretical diffraction Efficiency^b^	40%

^a^k refers to natural number including zero and any positive integer.

^b^Theoretical diffraction efficiency for a 2-levels FZL is 40%, calculated by scalar diffraction theory.

In Table 2, theoretical diffraction efficiency for a 2-levels FZL is 40% according to scalar diffraction theory^1^. The smallest linewidth is half of smallest pitch at 4.2 μm. Refractive index (n) of membrane material, measured by ellipsometer, was 1.66 at wavelength of 600 nm. Structure depth is relevant to refractive index and wavelength, and calculated to be odd times of 455 nm.

**Figure 5:**
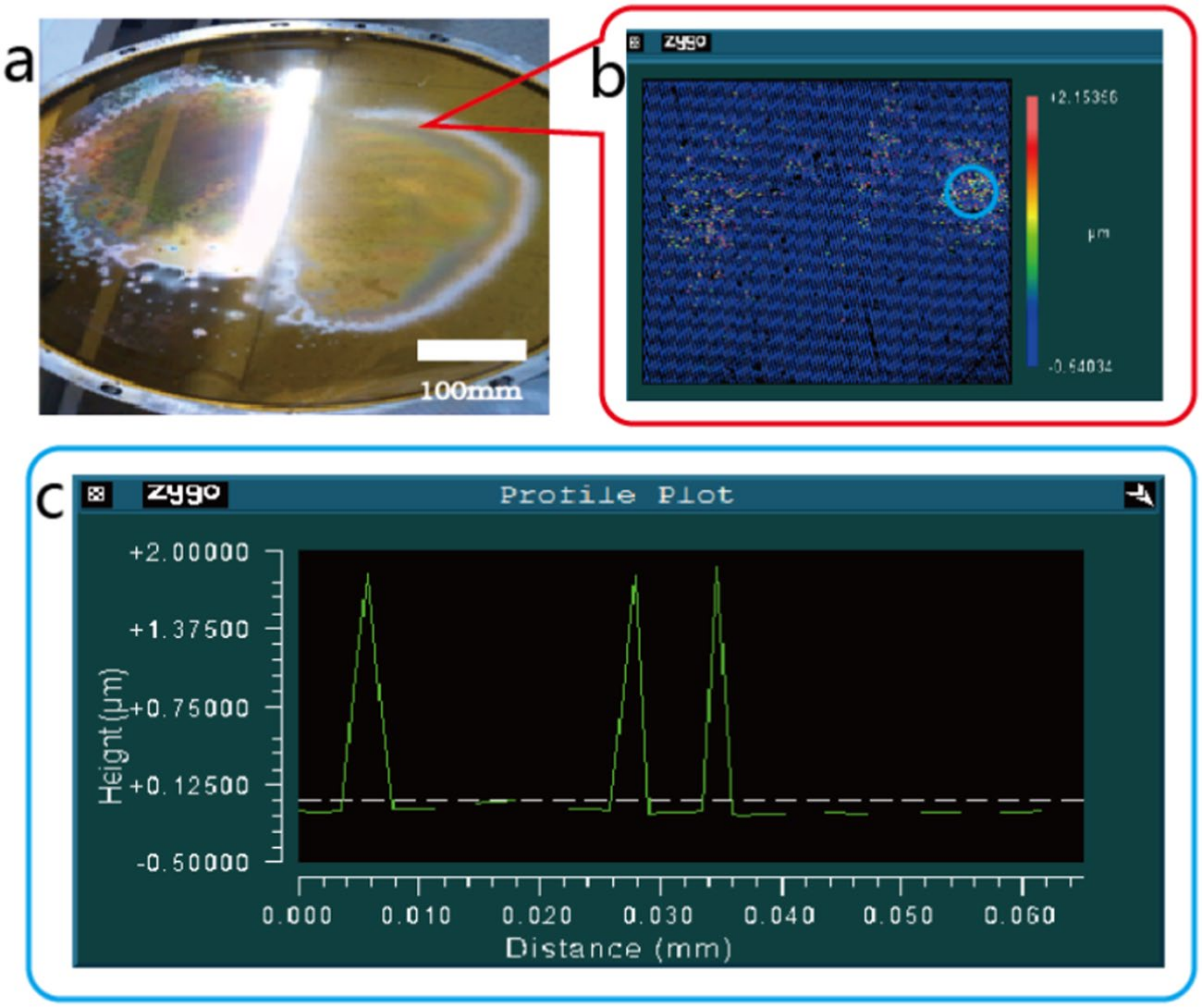
Macroscopic view of sample fabricated by conventional contact photolithography (**a**) and microstructure view of blurred area by Zygo white light interferometer (**b**), profile plot of recognized patterns within circled area whereas outside of that patterns are hardly recognized (**c**).

We fabricated a PI membrane off-axis FZL based on above optical parameters and aforementioned conventional contact photolithography method shown in Fig. 2. The result of conventional contact photolithography is shown in Fig. 5 below. The poor uniformity is clearly observed in macroscopic view of sample shown in Fig. 5(a) where some area is lack of diffraction effect and other area is uneven in color. Microscopic view of blurred area is observed through Zygo white light interferometer shown in Fig. 5(b) where patterns are unrecognized in most areas of observed spot. Profile plot of recognized patterns in blue circle area is shown in Fig. 5(c), steepness is calculated to be lower than 45 degrees and line to pitch ratio is lower than 25%.

**Figure 6:**
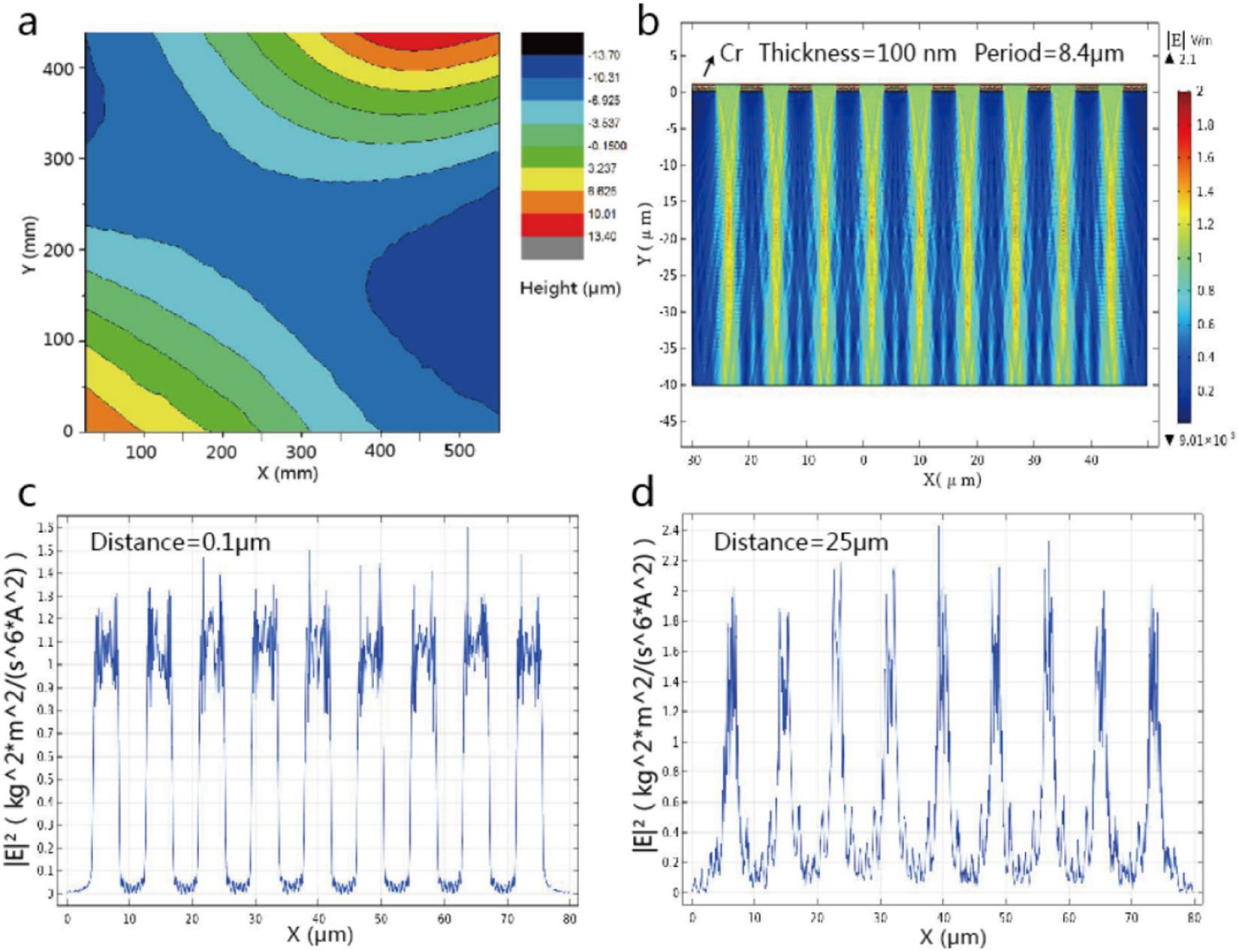
Flatness of large area photomask used in the experiment with PV around 27 μm within 450 mm × 550 mm full size (**a**), simulated light field distribution against distance from mask pattern layer where optical wavelength is 365 nm, grating period is 8.4 μm and Cr thickness is 100 nm (**b**), simulated light intensity at distance of 0.1 μm (**c**) and at distance of 25 μm (**d**).

The nominal flatness of high quality commercial large area photomask is around 25 μm in peak to value, the real flatness is measured around 27 μm depicted in Fig. 6(a) below through three coordinate measuring machine. According to Electromagnetic field theory, the light field distribution through Chromium grating varies with distance from mask pattern layer. We simulated light field distribution against distance from mask pattern layer using COMSOL software shown in Fig. 6(b) below. The Chromium (Cr) layer is 100 nm in thickness and 8.4 μm in period, and number of repetitive unit is 10. Optical wavelength is 365 nm in practice. We investigated how distance ranging from 0 to 40 μm influences light field distribution and plotted light intensity curves at distance of 0.1 μm and 25 μm shown in Fig. 6(c,d) respectively. It is obvious that light intensity distribution at distance of 0.1 μm to mask pattern is similar to ideal rectangular shape and theoretical resulted photoresist morphology would be standard grating. However, in practice light intensity distribution at distance of 25 μm to mask pattern is sharper and narrower. It is estimated that triangular shape photoresist morphology after development shown in Fig. 5(c) is caused by the distorted light field during UV exposure. Due to the poor flatness of large area photomask, large and non-uniform air gaps exist between photomask and PI membrane even if the back contact plate shown in Fig. 2 has a perfect flat surface shape.

To improve large area photoresist morphology, we need to decrease the value of air gaps and increase its uniformity. Vacuum assisted self contact method is put into practice and results are as follows.

### Vacuum assisted self contact photolithography

**Figure 7:**
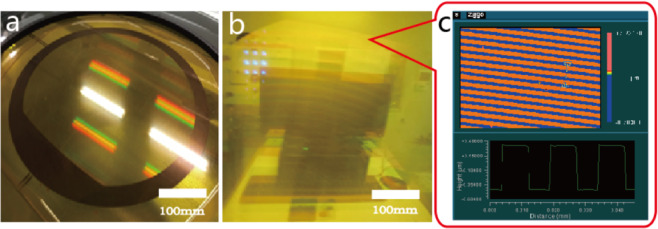
φ 400 mm aperture PI membrane in vacuum assisted self contact with off-axis FZL patterned photomask (**a**), macroscopic diffraction effect of the membrane off-axis FZL after development (**b**), profile plot of patterns at edge area (**c**).

We fabricated a PI membrane off-axis FZL based on vacuum assisted self contact photolithography method, and results are shown in Fig. 7 below.

**Figure 8:**
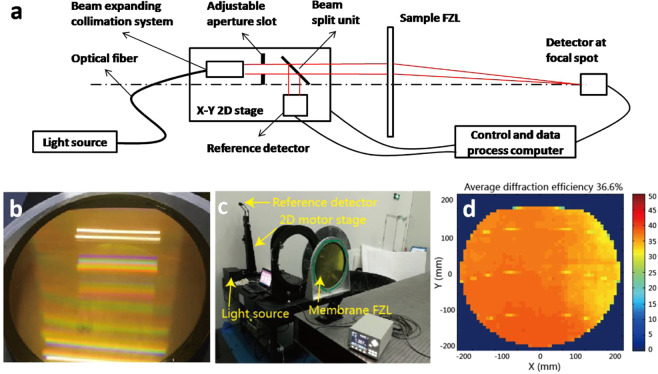
Diffraction efficiency measurement. Schematic of measurement device and principle (**a**), Photo of φ 400mm aperture PI membrane off-axis FZL (**b**), Photo of measurement site (**c**) and resulted average diffraction efficiency is 36.6% (**d**).

Uniform macroscopic diffraction effect of the membrane off-axis FZL shown in Fig. 7(b) indicates that structure morphology of photoresist is uniform in large area and profile plot of patterns at edge area shown in Fig. 7(c) verifies that point. To better know large area uniformity of structure morphology, we measured diffraction efficiency of the membrane off-axis FZL in full aperture, the result is shown in Fig. 8. As shown in Fig. 8(c) the membrane off-axis FZL is placed vertically in front of incident light and reference detector mounted on 2D motor stage, and detector of diffracted light is placed at focal spot 10.5 m away from membrane surface. The measurement system recorded light intensity of incident beam and diffracted beam, and calculated diffraction efficiency through Eq. (1) below.

1 $${\rm{\eta }}=\frac{{{\rm{I}}}_{{\rm{d}}}}{{{\rm{I}}}_{{\rm{i}}}}\times 100 \% $$

In Eq. (1) η stands for diffractive efficiency, I_d_ refers to light intensity of diffracted beam and I_i_ represents light intensity of incident beam. The calculated diffraction efficiency distribution is shown in Fig. 8(d) below with average diffraction efficiency of 36.6%. Because theoretical diffraction efficiency for 2 level FZL is 40%, thus over 90% of theoretical efficiency was obtained. It is possible to obtain much higher diffraction efficiency on 4-levels FZL in future through overlay process when we upgrade the method and device with submicron precision registration function.

The underlying mechanism of vacuum assisted self contact photolithography to achieve large area uniformity is discussed as follows. Looking at Fig. 3(b), an enclosed vacuum space is formed between photomask, silicone rubber sealing ring and PI membrane in which air pressure denotes as p_1_, volume denotes as V_1_, mole number denotes as n_1_ and temperature denotes as T_1_. According to State Equation of Ideal Gas, we have Eq. (2) shown below.

2 $${{\rm{p}}}_{1}{{\rm{V}}}_{1}={{\rm{n}}}_{1}{{\rm{RT}}}_{1}$$

Looking at Fig. 3(c), the enclosed vacuum space is compressed by atmosphere and reaches a new balance expressed in Eq. (3) below in which air pressure denotes as p_2_, volume denotes as V_2_, mole number denotes as n_2_ and temperature denotes as T_2_.

3 $${{\rm{p}}}_{2}{{\rm{V}}}_{2}={{\rm{n}}}_{2}{{\rm{RT}}}_{2}$$

Assume that there is no leakage or permeation through the enclosed space and no temperature variation thus n_1_ equals to n_2_, T_1_ equals to T_2_. Considering that cross sectional area of the enclosed space keeps unaltered, we can derive Eq. (4) below where d_1_ stands for air gap distance before contact which equals to thickness of silicone rubber sealing ring, d_2_ refers to air gap distance after contact. In the experiment, p_1_ equals to 5 Pa, p_2_ equals to 0.1 MPa, and d_1_ equals to 2 mm. By simple calculation we obtain the value of d_2_ expressed in Eq. (5).

4 $${p}_{1}{d}_{1}={p}_{2}{d}_{2}$$

5 $${d}_{2}=\frac{{p}_{1}{d}_{1}}{{p}_{2}}=100nm$$

We found that in theory air gap distance between photomask and membrane decreases to 100 nm after contact. In this way light intensity distribution in photoresist coating on membrane surface becomes standard rectangle shape shown in Fig. 5(c) and resulting photoresist morphology becomes uniform. If we further decrease thickness of silicone rubber sealing ring and improve vacuum degree, smaller air gap distance after contact can be achieved. This method takes advantage of membrane’s flexible feature and easy deformation capability. Theoretically, sub-nanometer air gap distance can be achieved by decreasing vacuum pressure below 0.05 Pa thus super high resolution is possible for photolithography. Additionally, it is also possible to apply vacuum assisted self contact photolithography on curved substrate in a way that firstly transfer patterns from silica photomask to membrane to form a membrane photomask, then transfer patterns from membrane photomask to curved substrate.

**Figure 9:**
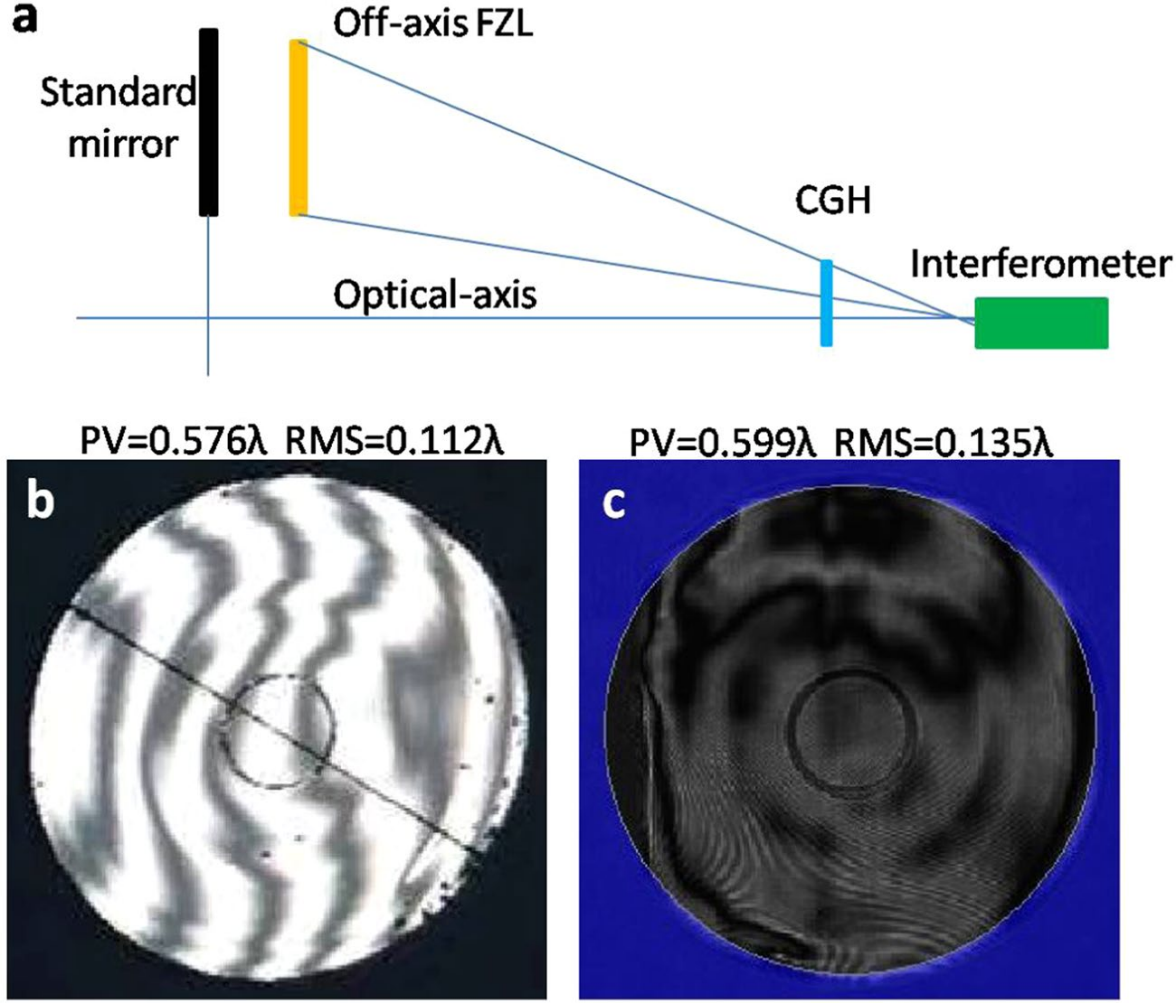
Schematic of membrane off-axis FZL wavefront error measurement setup (**a**), transmission wavefront of membrane substrate without pattern (**b**), and wavefront error of membrane off-axis FZL (**c**).

Furthermore, we characterized the imaging possibility of the membrane off-axis FZL. Wavefront error is an essential indicator of FZL imaging quality and schematic of off-axis FZL wavefront error measurement setup is shown in Fig. 9(a). The measurement principle is the same as the one applied to characterize smaller aperture on-axis FZL in our previous work^10^. Due to the poor optical uniformity of membrane substrate, transmission wavefront measured to be 0.576 λ peak to valley shown in Fig. 9(b), wavefront error of membrane off-axis FZL turn out to be 0.599 λ peak to valley shown in Fig. 9(c). It is important to point out that transmission wavefront of final FZL is dependent not only on pattern fabrication precision, but also on optical uniformity of membrane itself. Comparing the Fig. 9(b,c), there is no considerable deterioration on transmission wavefront after pattern fabrication. It is possible that we would achieve better imaging quality of membrane off-axis FZL if we can obtain membrane substrate with better optical uniformity.

## Conclusions

Optical polyimide membrane is a promising substrate material for diffractive lens applied in future large-aperture space based imaging system because of its light weight, environmental adaptability and deployable feature. In this letter, we put forward a facile large-area uniform photolithography technique using vacuum assisted self contact method to fabricate large-aperture membrane diffractive lens. We fabricated a φ 400 mm aperture membrane 2-levels off-axis Fresnel Zone Lens (FZL) based on the method and achieved uniformly distributed photoresist morphology as well as over 36.6% diffraction efficiency in full aperture. Imaging possibility of the membrane off-axis FZL was investigated. The results demonstrated that vacuum assisted self contact method effectively eliminates considerable air gaps caused by unevenness of large area photomask and substrate, thus facilitates uniform light field distribution in photoresist. This method takes advantage of membrane’s flexible feature and easy deformation capability. Theoretically, sub-nanometer air gap distance can be achieved by decreasing vacuum pressure below 0.05 Pa thus super high resolution is possible for photolithography. Additionally, the method is also possible to be applied on curved substrate in a way that firstly transfer patterns from silica photomask to membrane as a membrane photomask, and then transfer patterns from membrane photomask to curved substrate. This work provides reference to fabrication techniques of large aperture membrane diffractive lens, and offers feasible methods for future large area flexible electronics manufacturing.
